# Approaches to Predicting Outcomes in Patients with Acute Kidney Injury

**DOI:** 10.1371/journal.pone.0169305

**Published:** 2017-01-25

**Authors:** Danielle Saly, Alina Yang, Corey Triebwasser, Janice Oh, Qisi Sun, Jeffrey Testani, Chirag R. Parikh, Joshua Bia, Aditya Biswas, Chess Stetson, Kris Chaisanguanthum, F. Perry Wilson

**Affiliations:** 1 Yale University School of Medicine, New Haven, CT, United States of America; 2 Yale University School of Public Health, New Haven, CT, United States of America; 3 Program of Applied Translational Research, Yale University School of Medicine, New Haven, CT, United States of America; 4 Clinical Epidemiology Research Center, Veterans Affairs Medical Center, West Haven, CT, United States of America; 5 Helynx, Inc, Altadena, CA, United States of America; The University of Tokyo, JAPAN

## Abstract

Despite recognition that Acute Kidney Injury (AKI) leads to substantial increases in morbidity, mortality, and length of stay, accurate prognostication of these clinical events remains difficult. It remains unclear which approaches to variable selection and model building are most robust. We used data from a randomized trial of AKI alerting to develop time-updated prognostic models using stepwise regression compared to more advanced variable selection techniques. We randomly split data into training and validation cohorts. Outcomes of interest were death within 7 days, dialysis within 7 days, and length of stay. Data elements eligible for model-building included lab values, medications and dosages, procedures, and demographics. We assessed model discrimination using the area under the receiver operator characteristic curve and r-squared values. 2241 individuals were available for analysis. Both modeling techniques created viable models with very good discrimination ability, with AUCs exceeding 0.85 for dialysis and 0.8 for death prediction. Model performance was similar across model building strategies, though the strategy employing more advanced variable selection was more parsimonious. Very good to excellent prediction of outcome events is feasible in patients with AKI. More advanced techniques may lead to more parsimonious models, which may facilitate adoption in other settings.

## Introduction

Acute Kidney Injury (AKI)–an abrupt decline in kidney function–is a clinical condition that occurs in 10–20% of hospital admissions and remains the most common reason for inpatient nephrology consultation [1–6]. Patients who develop AKI have higher rates of morbidity, mortality, and end-stage kidney disease [7]. The incidence of AKI requiring renal replacement therapy (RRT) has increased over recent years, and over the past ten years the number of deaths associated with AKI requiring RRT has more than doubled [4]. The impact on the healthcare system is substantial as patients with AKI have longer length of stay (LOS) and double the hospital costs when compared to patients without AKI [8].

Early identification of high-risk patients would allow greater targeting of tailored interventions and more appropriate allocation of limited clinical resources [9]. Additionally, robust prognostic models would aid in the conduct of clinical trials by enriching the study population with individuals who are more likely to experience the clinical event of interest [10, 11]. Such models could also help in goals of care discussions. At present, few prognostic models exist to help physicians identify patients with AKI at risk of progression to RRT, increased mortality, or prolonged LOS, and the performance of existing prognostic models in AKI has been lackluster [12]. There are many reasons for this, ranging from the heterogeneity of AKI itself, to the patient populations used when developing prognostic models [2, 12–14]. In an effort to create conveniently applicable clinical prediction rules, several prognostic models have sacrificed accuracy for ease-of-use [13, 15]. In addition, few models use time-updated clinical data.

Conventional approaches to prognostic modeling rely on regression techniques including logistic and linear regression as well as Cox proportional hazards modeling [16–18]. These techniques have a long track-record, and are generally quite robust. However, they are prone to overfitting, and are limited in their ability to identify relevant interactions and nonlinearities. In addition, conventional statistical modeling is ill-equipped to handle the sheer number of potential covariates available in a modern electronic health record (EHR).

Due to the vast amounts of clinical data generated in the process of patient care, made easily accessible by the electronic health record (EHR), there has been increased interest in applying novel strategies to medical prognostic modeling [19, 20]. Several advanced modeling techniques used in the clinical setting to predict disease have shown enhanced accuracy for diagnosis when compared with regression methods [21–23]. Whether more advanced modeling approaches are superior to conventional approaches of model building in predicting outcomes of AKI remains unclear.

We sought to compare regression-based models to more advanced models to predict progression of AKI to RRT, death, or LOS in a time-updated manner. We hypothesized that the more advanced models would better prognosticate outcomes of AKI when compared to the conventional models in a validation cohort.

## Subjects and Methods

Detailed methods are provided as a supplement to this manuscript (S1 File).

Individuals in this study were enrolled in a randomized trial of an AKI alert system conducted at a single, large, urban tertiary care hospital (clinicaltrials.gov NCT01862419) [24, 25]. The protocol for this study was approved by the University of Pennsylvania Institutional Review Board. The original study was conducted under a waiver of informed consent as knowledge of participation in the study would invalidate patients randomized to the usual care group. The Institutional Review Board of the University of Pennsylvania Approved this consent procedure. All patients had AKI as defined by the Kidney Disease: Improving Global Outcomes creatinine criteria [26] After excluding patients whose diagnosis of AKI was based on a change from an outpatient creatinine value, we randomly split the dataset 1:1 into training and validation cohorts with the expectation that each cohort would be equally representative of the total study population.

Data extracted electronically from the EHR included all laboratory, medication, and procedural information as well as demographics and hospital discharge disposition. We constructed a modified Sequential Organ Failure Assessment (SOFA) score that did not include information regarding the Glasgow coma scale, as that covariate was unavailable [27].

### Conventional Model

We used backwards stepwise time-varying logistic regression (p-threshold 0.05) to model the outcomes of both dialysis within seven days and death within seven days. Candidate covariates included laboratory variables with at least one measurement in >95% of the training cohort which, after cubic spline creation, bore a significant relationship (p<0.05) to the outcome of interest in univariable models. The covariates included can be found in S1–S5 Tables. We also included demographic and medication information in the model building process. Medications were grouped *a priori* to indicate the prior receipt of: narcotics, paralytics, sedatives, antibiotics, and vasopressors. We clustered all models at the level of the individual patient.

We followed patients from the onset of AKI to discharge from the hospital or death. Risk predictions updated with each new medication, procedure order, or laboratory result, leading to a median of 128 (50–299) time-updated predictions per patient.

We used backwards stepwise linear regression (p-threshold 0.05) to model LOS using a variable selection approach identical to that described above. Patients who died in the hospital were excluded from this analysis. For the models predicting death and LOS, we used a two-equation approach, creating one model for time-points that occurred prior to the initiation of dialysis and one model for time-points after the initiation of dialysis, if any. The rationale for this approach is that certain covariates, particularly laboratory measurements, may be significantly altered by the process of dialysis rather than underlying physiological changes.

### Alternative Model

For the alternative model predicting dialysis, we used random forests to select independent covariates, which were ranked by their importance vectors [28]. To predict death and LOS, we used logistic regression on features extracted from principal components analysis of the lab values, and another set of principal components derived from medications data, the latter after being transformed with an exponential kernel to simulate the physical action of the drug [29]. Principal component analysis is an advanced model building technique that can summarize multidimensional correlated data and thus is suited for data sets with many variables such as an EHR [30, 31]. There were 386 medications taken into account in this modeling process.

Only the laboratory variables had any missing values. To account for this, we carried forward the most recently measured laboratory variable for each participant at any given time point. If no prior lab value was available, we assigned the median value in the training cohort.

We assessed model discrimination using c-statistics for the binary outcomes, and the R^2^ value for LOS. C-statistics were compared using the SomersD package in Stata, accounting for clustering of values within individual patients [32]. We performed all analyses in Stata v. 14.0 (StataCorp, College Station, TX) and via proprietary software developed by Helynx, Inc (Altadena, Ca).

## Results

### Baseline Characteristics

Of 2393 individuals in the full acute kidney injury (AKI) cohort, we excluded 152 (6.4%) patients whose diagnosis of AKI was dependent upon an outpatient creatinine value. The remaining individuals were randomly split into the training (n = 1,098) and validation (n = 1,143) cohorts. Baseline characteristics of the two cohorts appear in Table 1. The mean (SD) age in years was 62.1 in the training cohort and 62.8 in the validation cohort. 56.1% of the training cohort population was male, and 26.8% identified as black while 55% of the validation cohort was male and 27% identified as black. The groups were similar in their common comorbidities with congestive heart failure occurring in 32.4% of the training cohort and 32.7% of the validation cohort, and diabetes occurring in 27.9% of the training cohort and 32.2% of the validation cohort. CKD occurred in 25.4% of the training cohort and 26.9% of the validation cohort.

**Table 1:** Baseline Characteristics at the Onset of AKI^1^.

	Training Cohort (n = 1098)	Validation Cohort (n = 1143)	P-Value
*Demographics*			
Age (yr)	62.1 (51.7–71.7)	62.8 (51.8–71.7)	0.85
Male Sex (%)	617 (56.3)	628 (55.5)	0.68
Black (%)	294 (26.8)	309 (27.0)	0.89
Hispanic (%)	28 (2.6)	37 (3.3)	0.33
BMI	28.0 (24.0–33.0)	27.0 (23.0–32.0)	0.02
*Laboratory Data*			
Anion Gap, per 1 unit	8.0 (7.0–10.0)	8.0 (6.0–10.0)	0.95
Bicarbonate, meq/L	24.0 (22.0–27.0)	25.0 (22.0–27.0)	0.87
BUN, mg/dL	21.0 (12.0–32.0)	21.0 (13.0–33.0)	0.54
BUN Slope, mg/dl/24h	4.7 (0.9–9.7)	4.5 (0.9–9.8)	0.98
Calcium, mg/dL	8.4 (7.9–8.9)	8.4 (7.9–8.9)	0.96
Chloride, meq/L	104.0 (101.0–108.0)	104.0 (100.0–108.0)	0.97
Creatinine, mg/dL	1.4 (1.0–1.8)	1.4 (1.0–1.9)	0.39
Creatinine Slope, mg/dl/24h	0.4 (0.2–0.7)	0.4 (0.2–0.7)	0.69
Glucose, mg/dL	123.0 (100.0–156.0)	122.0 (98.0–156.0)	0.48
Hematocrit, %	31.0 (27.0–35.0)	30.0 (27.0–35.0)	0.17
Hemoglobin, g/dL	10.2 (8.9–11.7)	10.1 (8.9–11.5)	0.10
Magnesium, meq/L	2.0 (1.8–2.2)	2.0 (1.8–2.2)	0.83
Mean Corpuscular Hemoglobin (MCH), pg/cell	30.1 (2.8)	30.1 (2.9)	0.63
MCH Concentration, g/dL	33.0 (32.0–34.0)	33.0 (32.0–34.0)	0.16
Platelet Count, 1000/uL	185.0 (117.0–263.0)	189.0 (131.0–261.0)	0.26
Potassium, meq/L	4.2 (3.8–4.6)	4.2 (3.8–4.6)	0.50
Red Cell Distribution, %	15.6 (14.2–17.4)	15.9 (14.4–18.0)	0.002
Sodium, meq/L	137.0 (135.0–140.0)	137.0 (134.0–140.0)	0.92
White Blood Cell Count, 100/uL	9.5 (6.5–13.9)	9.7 (6.6–14.4)	0.25
Pantoprazole Use^3^, %	51 (4.6%)	54 (4.7%)	0.93
*Comorbidities*			
CHF^2^	356 (32.4)	375 (32.9)	0.82
Diabetes	309 (28.1)	368 (32.2)	0.04
Cancer	294 (26.8)	288 (25.2)	0.41
Chronic Kidney Disease	279 (25.4)	307 (26.9)	0.42
Liver Disease	164 (14.9)	158 (13.8)	0.45

Baseline characteristics at AKI onset. Comparisons between continuous covariates were made with rank-sum tests, and categorical covariates with chi-square tests.

^1^ AKI = Acute Kidney Injury as defined by KDIGO creatinine criteria.

^2^ CHF = Congestive Heart Failure.

^3^Pantoprazole was the only proton-pump inhibitor on formulary at the hospital at the time of this study.

AKI onset occurred at a median (IQR) of 2.7 (1.4–6.0) days after hospitalization. Among the 169 (7.5%) of patients who received dialysis, the median time from AKI to dialysis was 2.3 (0.74–6.2) days. There were 220 total deaths (9.8%), and the median length of stay was 10.2 (6.0–17.2) days.

### Prediction of Dialysis

#### Conventional Model

Our model predicting dialysis within seven days appears as S1 Table. Of 21 considered covariates, we were left with a model containing 8 significant covariates after backwards stepwise regression. Three of these covariates were directly related to renal function (serum creatinine, increasing serum creatinine, and slope of BUN).

#### Alternative model

The random forests model selected 2 continuous covariates and 1 categorical covariate. Continuous covariates included the mean and slope (calculated using linear regression of 3 days) of the serum creatinine, and the categorical covariate was prior pantoprazole use.

#### Comparison

**Fig 1:**
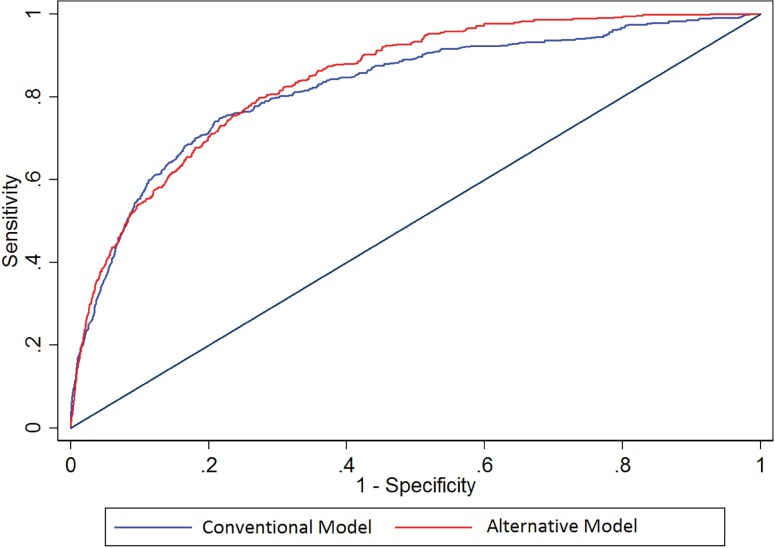
Receiver-Operator Characteristic curves for Dialysis. Receiver-operator characteristic (ROC) curves comparing the performance of conventional vs. alternative models in the prediction of dialysis in the validation cohort. Area under the curve for conventional model: 0.82 (0.76–0.88), alternative model 0.84 (0.80–0.89).

The conventional and alternative models had similar excellent abilities to predict dialysis within 7 days (Table 2, Fig 1), p-for comparison = 0.28. The area under the curve (AUC) was 0.82 for the conventional model and 0.84 for the alternative model.

**Table 2:** Comparison of prognostic models in the validation cohort.

Outcome	Conventional Model, Training Cohort	Alternative Model, Training Cohort	Conventional Model, Validation Cohort	Alternative Model, Validation Cohort
**Dialysis, AUC**^**1**^	0.89 (0.86–0.93)	0.88 (0.86–0.90)	0.82 (0.76–0.88)	0.84 (0.80–0.89)
**Death, AUC**^**1**^	0.90 (0.88–0.93)	0.85 (0.81–0.90)	0.80 (0.75–0.84)	0.80 (0.76–0.85)
**Length of Stay**^**2**^**, R**^**2**^	0.44 (0.32–0.57)	0.26 (0.21–0.30)	0.17 (0.10–0.24)	0.20 (0.14–0.26)*^3^

^1^ Death and dialysis are evaluated in terms of area under the receiver-operator characteristic curve (AUC).

^2^ Length of stay is evaluated in terms of the model R^2^.

^3^ * = p<0.05 compared to conventional model.

### Prediction of Death

#### Conventional Model

Many more laboratory factors were significantly associated with death within 7 days than with dialysis within 7 days, leaving 50 candidate covariates to be included in the multivariable model (S2 Table). After backwards stepwise regression, we were left with a pre-dialysis prognostic model containing 14 covariates and a post-dialysis prognostic model containing 13 covariates. Covariates shared between the models included surgical patient status (though this was protective pre-dialysis and harmful post-dialysis), anion gap, hemoglobin (higher levels protective pre-dialysis, harmful post-dialysis), potassium, and sodium.

#### Alternative Model

**Fig 2:**
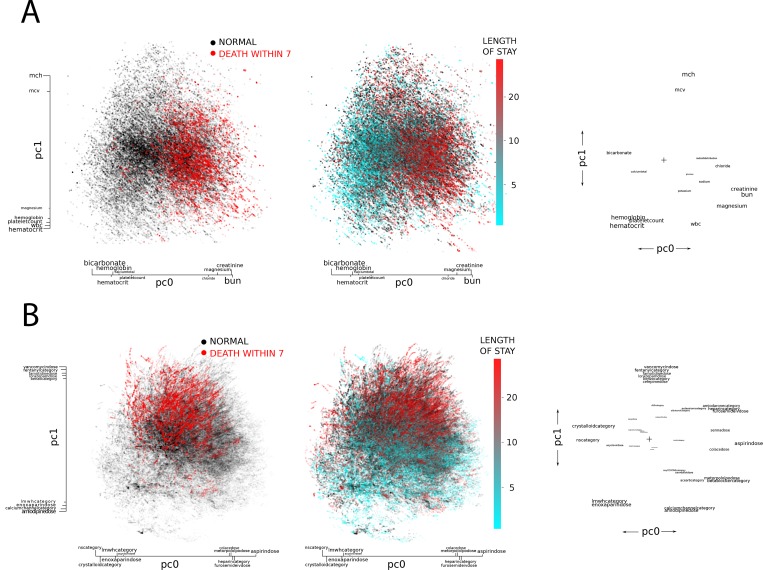
Principal Components Analysis. Colored points reflect individual level data, where individuals are mapped to a coordinate plane based upon 2 principal components derived from laboratory (panel A) and medication (panel B) data. Next to the colored plots, the covariate map appears. Covariates are mapped along the same two principal component vectors, helping to illustrate the correlations among several of the covariates. **A)** Laboratory covariates as mapped on two principal components. Based on laboratory values, a patient (represented as a dot) can be put anywhere on the coordinate plane. For the outcome of death within 7 days, red dots indicate an individual who died in that time frame, black an individual who did not. For LOS analyses, blue dots indicate shorter lengths of stay, with red dots indicating longer lengths of stay. Clustering of colors along one dimension of the plot suggests a significant relationship between that principal component and the outcome. Next to the patient plots is a plot showing each lab on the same two principal coordinate axes. Labs that are closer together a more correlated (for example, creatinine and BUN). Size of the text indicates strength of association between a given lab and that principal component. **B)** Medication covariates as mapped on two principal components. Based on medications received, a patient (represented as a dot) can be put anywhere on the coordinate plane. For the outcome of death within 7 days, red dots indicate an individual who died in that time frame, black an individual who did not. For LOS analyses, blue dots indicate shorter lengths of stay, with red dots indicating longer lengths of stay. Clustering of colors along one dimension of the plot suggests a significant relationship between that principal component and the outcome. Next to the patient plots is a plot showing each medication on the same two principal coordinate axes. Medications that are closer together a more correlated (for example, vancomycin and fentanyl). Size of the text indicates strength of association between a given lab and that principal component. Covariates ending in "category" are binary (ie D50 category is a 1 if the patient has received 50% dextrose infusion), whereas those ending in "dose" reflect the actual dose received. Higher resolution figures are available in S2 File.

After principal components analysis, three principle components were significantly associated with death, with 1 derived from laboratory data and 2 derived from medication data. Graphs of these principal component arrays and the outcomes of interest appear as Fig 2A and 2B. The laboratory principal component axis was defined by higher levels of creatinine, BUN, and chloride, and by lower levels of bicarbonate, hemoglobin, and platelets. In terms of medication principal components, one axis appeared to segregate along medications associated with volume status (with the margins of the axis defined by crystalloid infusion on one end and furosemide on the other), and one axis that connoted clinical severity (with fentanyl, vancomycin, cefepime on one end and amlodipine and low-molecular weight heparin on the other).

#### Comparison

**Fig 3:**
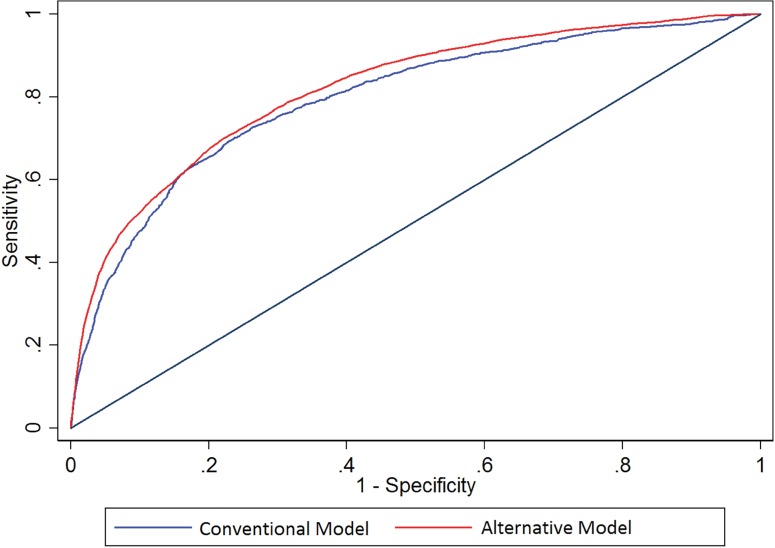
Receiver-Operator Characteristic curves for death. Comparing the performance of conventional vs. alternative models in the prediction of death in the validation cohort. Area under the curve for conventional model: 0.80 (0.75–0.84), alternative model 0.80 (0.76–0.85).

The conventional and alternative models had similar, very good abilities to predict death within 7 days (Table 2, Fig 3), p-for comparison = 0.60. The AUC was 0.80 for the conventional model and 0.80 for the alternative model. The SOFA score had good prognostic ability in this setting with AUC 0.75 (0.70–0.81). At a p-value threshold of <0.05, this was statistically worse than the alternative model (p = 0.04) but not the conventional model (p = 0.06).

### Prediction of Length of Stay

#### Conventional Model

From an initial pool of 50 candidate covariates, our stepwise linear regression model selected 12 that were significantly associated with LOS prior to the initiation of dialysis, and 7 that were significantly associated with LOS after the initiation of dialysis. Factors that appeared in both models included platelet count (higher count associated with shorter LOS) and the use of total parenteral nutrition (associated with longer LOS in both models) (S4 Table).

#### Alternative Model

The same principle component vectors identified in the prognostication of death were applied to predict length of stay (Fig 2A and 2B).

#### Comparison

In terms of length of stay prognostication, the alternative model outperformed the conventional model (r^2^ 0.20 vs. 0.17, p = 0.048). Both models significantly outperformed the modified SOFA score (r^2^ 0.07, p<0.001 for both comparisons).

## Discussion

When treating patients with AKI, a successful prognostic tool would ideally utilize readily available clinical data, easily update during a patient’s hospital stay as new data becomes available, and accurately reflect the risk of outcomes that are of clinical interest. Both conventional and alternative approaches can accommodate these conditions, but it is unclear whether alternative methods would offer substantial advantages over conventional methods. In this study, we found that alternative models performed as well as or better than conventional prognostic modeling in a prospectively collected AKI dataset.

The covariates selected by both modeling approaches were not particularly surprising. The prediction of dialysis was heavily dependent on factors associated with renal function such as creatinine and BUN, while the predictors of death and length of stay were primarily proxies of illness severity. Prior studies have also identified these factors as being of prognostic interest [13, 14, 18, 33]. Interestingly, the random forest algorithm selected pantoprazole use as a noteworthy feature of model building. Proton-pump inhibitor use has been associated with AKI in several prior studies demonstrating that hypothesis-agnostic algorithms may reveal clinically meaningful drug-organ interactions [34, 35].

Conventional statistical approaches have difficulty analyzing the vast space of possible medication use (hence our need to classify important medication categories *a priori* in the conventional models, but not in alternative models). Principal component analysis on the other hand may be may be particularly useful as a form of modeling that can employ a large panel of covariates as in the case of the EHR.

Our conventional modeling approach was not straightforward. It involved variable transformations in the form of splines as well as stepwise regression to obtain a parsimonious model. As such, the results of our conventional modeling may be considered a best-case scenario. The ability of the alternative models to incorporate a wider breadth of covariates in model building is particularly well-suited to EHR applications, and may be easier to implement. As the data in EHRs accumulates, alternative models may be better equipped to handle the scope of data, including multiple covariates as well as the scope of medication use, than conventional approaches. Further, the computational infrastructure of the EHR allows for more complex modeling, as probabilities of outcome can be computed *in silico* and presented to the end-user. This obviates the need for simple risk scores that have dominated prognostic modeling in the past.

The results of this study should be interpreted in light of several limitations. We limited our model building to include variables that we felt would be present in most EHRs. As such, we excluded data on comorbidity, which may not be updated in real-time during a hospitalization. This may have decreased the performance of both the conventional and alternative models. In addition, the data come from a single health system–the validation was internal, based on a withheld half of the dataset. External validation would be impossible given the vast number of covariates considered for inclusion; the methods utilized in our alternative models are optimal when applied to the health record within a single health system.

Despite these limitations, our study demonstrates that we can readily apply novel model building strategies to EHR data in order to make clinically relevant predictions. Furthermore, we have shown that time-updating of risk scores is feasible in the context of AKI. Future work in this area should focus on real-time risk prediction at both the individual institutional and multi-institutional levels. There is also a need for interventional studies that examine the use of risk modeling to benefit individual patients; such studies could evaluate more robust targeting of electronic alert systems for AKI.

## Supporting Information

S1 File Extended Methods. (DOCX)

S2 File High Resolution Images. (ZIP)

S1 Table Conventional Model Predicting Dialysis. (DOCX)

S2 Table Conventional Model Predicting Death, Death ORs Before Dialysis Initiation. (DOCX)

S3 Table Death OR’s, Before Dialysis Initiation. (DOCX)

S4 Table Traditional Model Predicting Length of Stay. (DOCX)

S5 Table Length of Stay After Dialysis Initiation. (DOCX)
