# Supplementary material for: Approaches to Predicting Outcomes in Patients with Acute Kidney Injury
Source: PLoS One. 2017 Jan 25;12(1):e0169305. doi: 10.1371/journal.pone.0169305 (PMC5266278; doi:10.1371/journal.pone.0169305)
Supplement: S1 File — (DOCX) [file pone.0169305.s001.docx]

*Cohort Development*

We considered for analysis all patients enrolled in the AKI-Alert study (methods previously published), a randomized, controlled trial evaluating the efficacy of an electronic text-paging alert system for AKI among hospitalized patients in a single, urban, tertiary care center.^1,2^ In that study, AKI was defined using the Kidney Disease: Improving Global Outcomes consensus creatinine criteria.^3^ We excluded individuals whose diagnosis of AKI was based only upon a change from an outpatient creatinine value.

*Covariate Ascertainment*

Data extracted electronically from the EHR included all laboratory, medication, and procedural information as well as demographics and hospital discharge disposition. We constructed a modified Sequential Organ Failure Assessment (SOFA) score which did not include information regarding the Glasgow coma scale as that covariate was unavailable.^4^

*Traditional Prognostic Model Creation*

Our goal was to create models that could utilize time-updated clinical data to estimate the probability of a given outcome. Our primary outcomes were: dialysis within 7 days, death within 7 days, and length of stay. As such, the risk score for any given patient would update throughout the hospitalization. Such a system could be used to risk stratify individuals at any point during hospitalization, and could be considered as enrollment criteria for future clinical trials.

We took a semi-unbiased approach to variable selection for modeling. Modeling was performed exclusively in the training cohort. All laboratory variables that were measured at least once in >95% of individuals in the training cohort were considered for inclusion (see supplementary tables for model covariates). We also considered specific demographic, medication, and geographic covariates (eg ICU versus non-ICU) based on our prior research and clinical intuition.^5^ We did not include comorbidity information in modeling despite the availability of this data, as comorbidity information may not always be available in real time.

Given the different number of observations per patient, we compared a standard discrete-time logistic regression model to a random-effects logistic regression model with internal validation within the derivation cohort. The models performed nearly identically for all covariates examined suggesting that significant patient-level effects do not modify the risk of categorical outcomes in terms of the factors analyzed.

For the dialysis model, development was based on all data from the onset of AKI to discharge or 24-hrs prior to the initiation of dialysis. We excluded data in the 24-hrs prior to initiation of dialysis to avoid biasing our model towards factors that become acutely perturbed immediately prior to dialysis but might be less useful clinically (as our interest is in predicting dialysis in the near, but not immediate, future). For the model predicting death, we utilized all data from the onset of AKI to hospital discharge. For length of stay, we utilized all data from the onset of AKI to discharge or 30-days later. For death and LOS, we created two predictive models, one that applied to time prior to the initiation of dialysis and one that applied to time after the initiation of dialysis, as the impact of covariates on death (particularly laboratory covariates) may change significantly after dialysis is initiated.

For all models, we created restricted cubic spline plots for all eligible laboratory variables and visually inspected them for non-linearity and for reasonable cut-points. Cubic spline plots were used as many laboratory variables may display non-linear relationships with outcomes. To determine whether cubic spline creation was worthwhile, we compared the significance of laboratory predictors with the outcome of interest in univariable analysis using likelihood ratio tests. If a linear model was as strongly associated with outcome as the spline model, the simpler (linear) model was used. We evaluated univariable associations between covariates and outcomes using Wilcoxon Rank-Sum tests or chi-square tests as appropriate. Lab values with no significant relationship to outcome after splining were not included in multivariable models. Laboratory values, demographic information, ICU location, surgical status, and time from AKI onset were included in a multivariable model. For death and dialysis, this model used logistic regression and we applied backwards stepwise logistic regression with a p-threshold of 0.05 to create the final models as documented in Supplemental Tables 1 and 2. For LOS, we applied backwards stepwise linear regression with a p-threshold of 0.05 to create the final multivariable model. All models were clustered at the level of the patient to account for non-independence of measures.

*Machine Learning Model Creation*

We conditioned the underlying data using the following procedures: The top 35 most frequently delivered drugs and the top 16 most frequently recorded analytes were chosen. These analyte data were then transformed using a box-cox (power law) transformation.

Drug delivery events were conditioned according to the following rule: A drug delivery event was followed by an exponentially decaying signal with a time constant proportional to the median delivery interval for that drug calculated across the training data. Successive exponential decays for repeat drug deliveries sum linearly. These signals were then transformed using a box-cox transformation.

For the dialysis outcome, the primary algorithm used was simple discrete logistic regression, informed by random forest classification^6^, random forest classifiers were trained on the data, and the most important features were selected for inclusion into a final logistic regression model. The rationale was that random forest classifiers are more likely to identify uncorrelated variables, while logistic regression is more likely to generalize. The final features used were a single continuous feature, creatinine, a single moment (change in creatinine in time), and a single categorical variable (delivery of pantoprazole).

We used the following procedure to identify a categorical variable which would be independent of the continuous variables used in prediction, Cre and dCre/dt. From among the top five categorical features which had the highest feature weights from random forests, we sorted by an additional metric designed to highly weight predictors of dialysis soon after admission: the ratio of patients identified by the feature receiving dialysis within 5 days of admission to those receiving dialysis after more than 5 days. That metric came out as follows: pantoprazole: 0.80, cefepime: 0.41, phenylephrine: .2, total enteral nutrition: 0.18, total parenteral nutr 0.0. We chose the highest ranked categorical feature for the model we submitted to the validation set.

Features for the final Random Forest classification algorithm were selected through a 5-fold cross-validation process: for each of 5 partitions of the training set (all data points associated with a given patient were required to be in the same partition), the algorithm was trained on the remainder of the data, then used to determine classification probabilities for data points in that partition. After an initial step of minimum redundancy maximum relevancy^7^ selection to eliminate clearly irrelevant features, we iterated through combinations of features with this 5-fold cross-validation process, optimizing for the highest classification performance (using the c-statistic) with a relatively small number of features. We also tested other classifiers, including regularized logistic regression, nearest-neighbors and support-vector machine classifiers, but these did not perform as well in internal cross-validation.

For the death and length-of-stay outcome, we subjected all transformed analytes and drug data to principal components analysis. Whereas analytes are recordings of patient condition, drugs represent decisions made by caregivers. We therefore elected to keep the drug and analyte principal components' calculation separate, reasoning that they represented independent dimensions of the care spectrum.

Following principal components analysis, the principal components and projections of the data onto these principal components were visually inspected, colored by the quantities of interest (death within 7 days, length-of-stay; see Figure 2). We noticed that only the first analyte-derived principal component contained variance relevant to the quantities of interest, whereas both the first two drug-derived principal components had meaningful variance. The projection of the data onto these three principal components formed the basis for our regressions. Death within 7 days was classified using a logistic regression, and length-of-stay was fit using a linear regression.

Feeding higher-order principal components to the regressions eroded performance. Categorical variables that were delivered once per patient were also considered, and were conditioned similarly to the analyte values, but due to their binary nature did not conform to multidimensional gaussian distributions well-suited to PCA, and did not produce principal components that contributed significantly to the training accuracy.

In addition to the linear methods mentioned, we tried the following additional classification procedures to classify death-within-7 days: random forests with 50 trees, naive bayes using a gaussian kernel, and support vector machines using a radial basis function kernel. On cross-validation within the training data sets, these methods did not perform as well as principal component analysis.

*Missing Data*

Only laboratory values had any missingness in either the training or validation datasets. Both the traditional and machine-learning approach applied the same technique to account for missing data, which was to carryforward the last laboratory value into future time-points if a prior value was available. If not, the value was imputed to be the median of all the values in the training data set.

*Model Validation*

In the validation cohort, we calculated risk scores for dialysis, death, and length of stay using both the traditional and machine-learning models. We assessed model performance in the validation cohort using c-statistics in the case of dialysis and death outcomes, and r^2^ values in the case of the LOS outcome. We created C-statistic confidence intervals accounting for clustering within individuals using the “somersd” package in Stata.^8^ We compared C-statistics using the method described by Delong et al.^9^ We calculated R^2^ confidence intervals through a 50-fold bootstrap procedure and made comparisons between R^2^ values using a Fischer’s z transformation.^10^

All traditional analyses were performed in Stata v. 14.0 (StataCorp, College Station, TX). All machine-learning analyses were performed by Helynx, inc (Altadena, Ca) using their proprietary software.

1. Wilson FP, Reese PP, Shashaty MG, et al. A trial of in-hospital, electronic alerts for acute kidney injury: design and rationale. *Clin. Trials.* 2014:1740774514542619.

2. Wilson FP, Shashaty M, Testani J, et al. Automated, electronic alerts for acute kidney injury: a single-blind, parallel-group, randomised controlled trial. *The Lancet.* 2015;385(9981):1966-1974.

3. Kidney Disease: Improving Global Outcomes (KDIGO) Acute Kidney Injury Work Group. KDIGO Clinical Practice Guideline for Acute Kidney Injury. *Kidney Int.* 2012;2(Suppl.):1-138.

4. Vincent JL, Moreno R, Takala J, et al. The SOFA (Sepsis-related Organ Failure Assessment) score to describe organ dysfunction/failure. On behalf of the Working Group on Sepsis-Related Problems of the European Society of Intensive Care Medicine. *Intensive Care Med.* 1996;22(7):707-710.

5. Wilson FP, Yang W, Feldman HI. Predictors of death and dialysis in severe AKI: the UPHS-AKI cohort. *Clin. J. Am. Soc. Nephrol.* 2013;8(4):527-537.

6. Breiman L. Random forests. *Machine learning.* 2001;45(1):5-32.

7. Peng H, Long F, Ding C. Feature selection based on mutual information criteria of max-dependency, max-relevance, and min-redundancy. *Pattern Analysis and Machine Intelligence, IEEE Transactions on.* 2005;27(8):1226-1238.

8. Newson R. Confidence intervals for rank statistics: Somers' D and extensions. *Stata Journal.* 2006;6:309-334.

9. DeLong ER, DeLong DM, Clarke-Pearson DL. Comparing the areas under two or more correlated receiver operating characteristic curves: a nonparametric approach. *Biometrics.* 1988:837-845.

10. Cox NJ. Speaking Stata: Correlation with confidence, or Fisher’sz revisited. *Stata J.* 2008;8:413-439.
