# Supplementary figures and images for: Approaches to Predicting Outcomes in Patients with Acute Kidney Injury
Source: PLoS One. 2017 Jan 25;12(1):e0169305. doi: 10.1371/journal.pone.0169305 (PMC5266278; doi:10.1371/journal.pone.0169305)

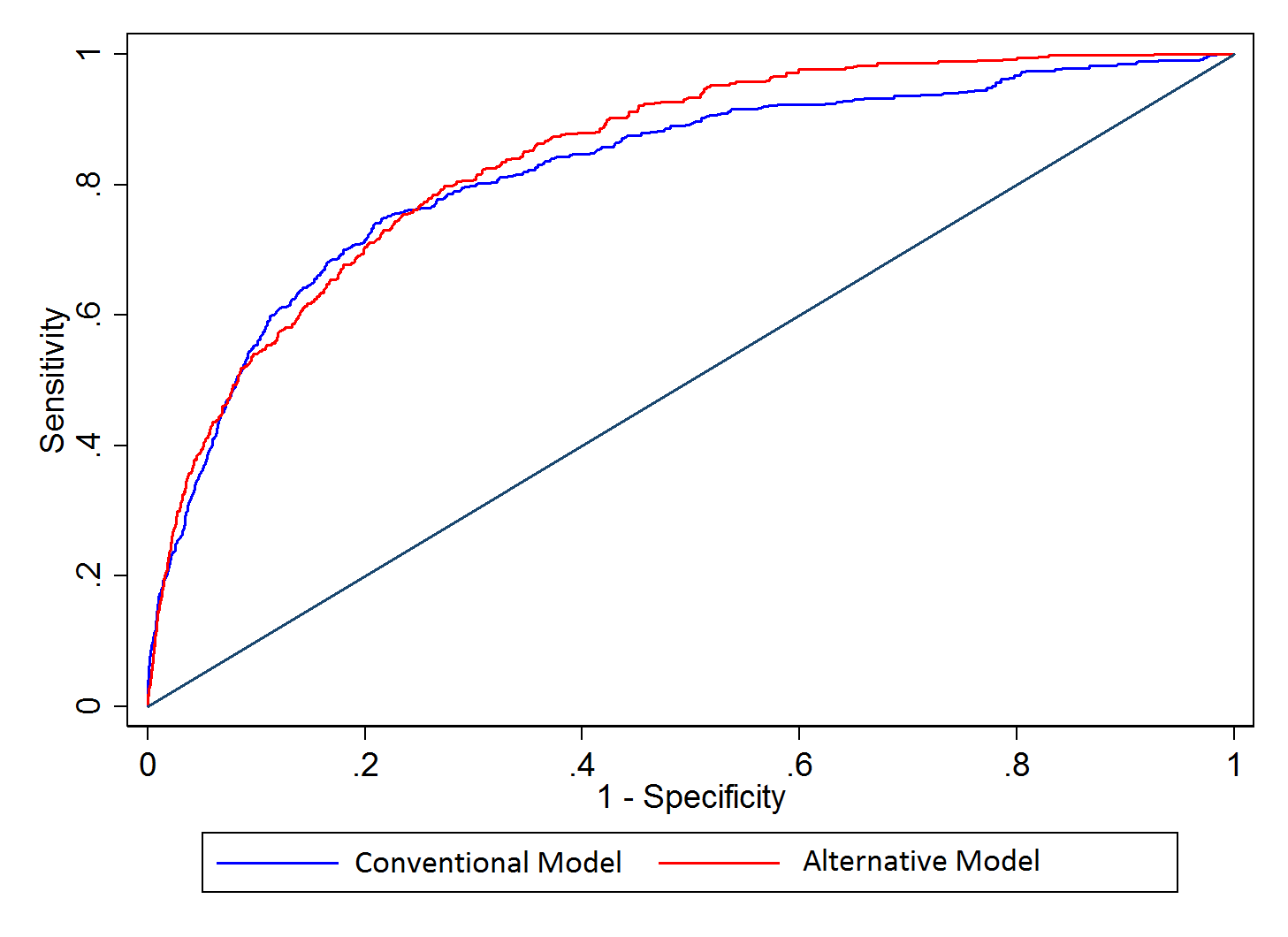

Supplement: S2 File — (ZIP) [file pone.0169305.s002.zip › fig1.png]

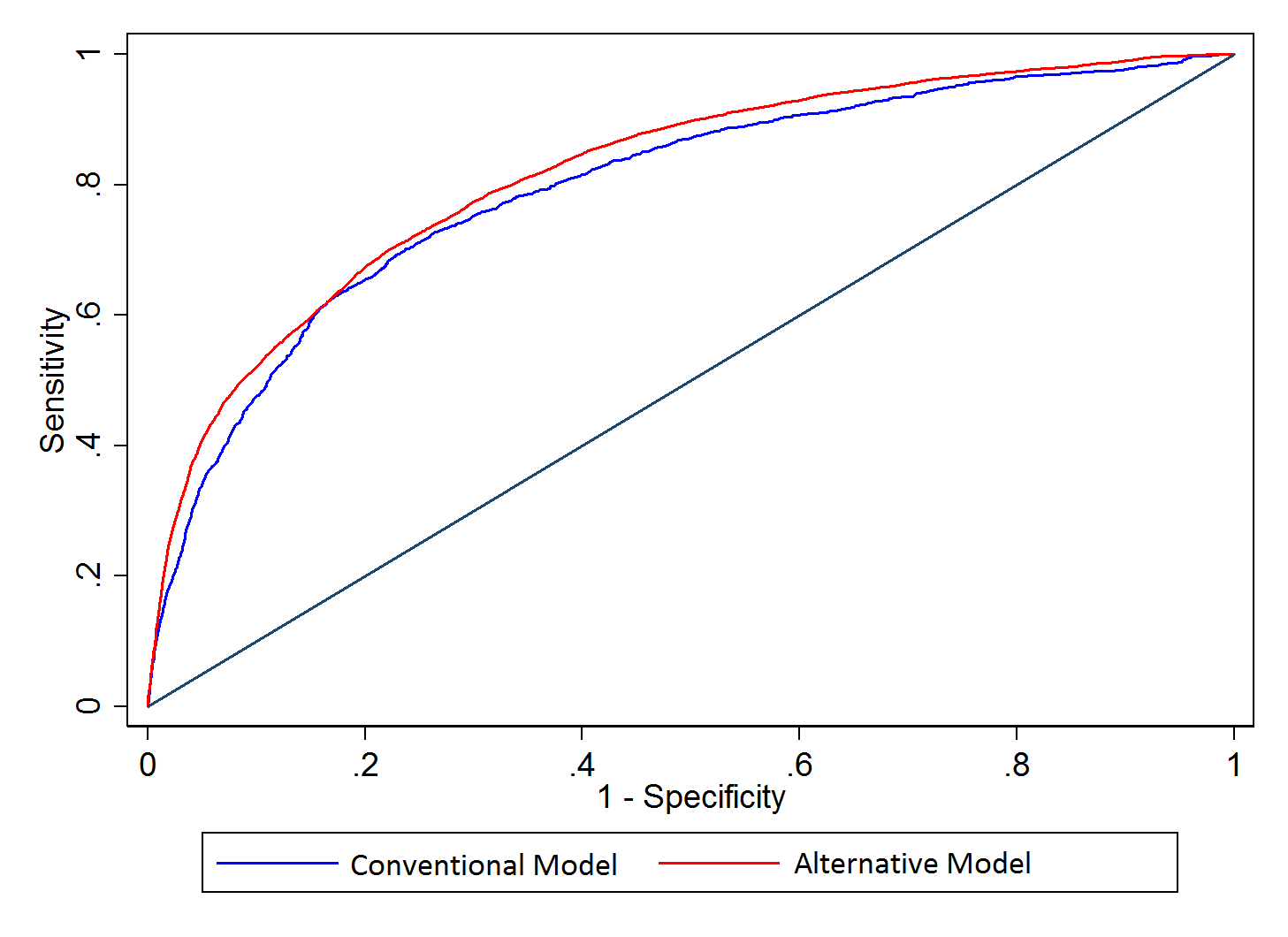

Supplement: S2 File — (ZIP) [file pone.0169305.s002.zip › fig3.png]

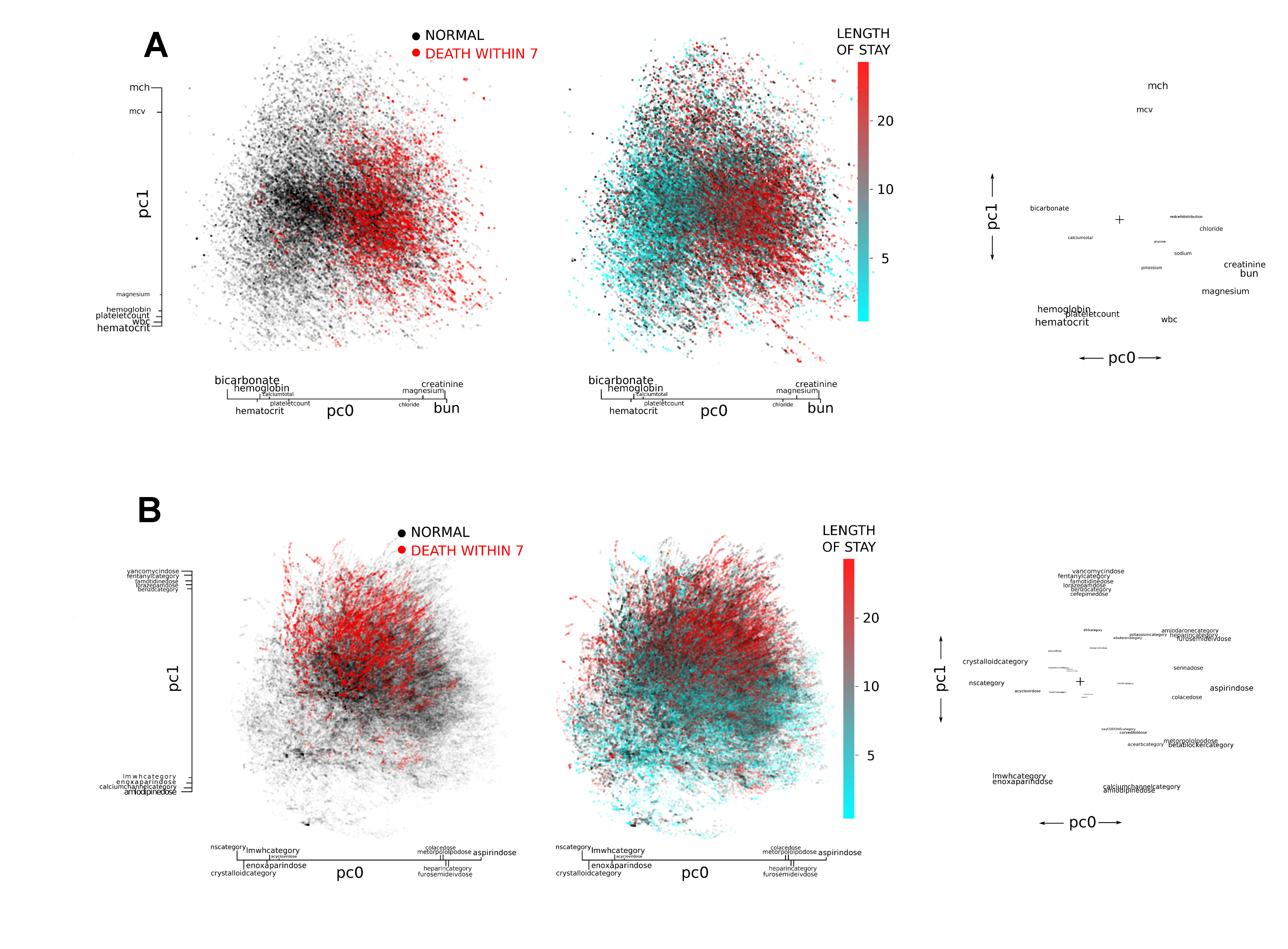

Supplement: S2 File — (ZIP) [file pone.0169305.s002.zip › fig2.bmp]
