# Supplementary material for: Approaches to Predicting Outcomes in Patients with Acute Kidney Injury
Source: PLoS One. 2017 Jan 25;12(1):e0169305. doi: 10.1371/journal.pone.0169305 (PMC5266278; doi:10.1371/journal.pone.0169305)
Supplement: S1 Table — (DOCX) [file pone.0169305.s003.docx]

**S1 Table – Conventional Model Predicting Dialysis**

| **Covariate^1^** | **Univariable OR^2^ (95% CI)** | **Full Multivariable Model OR (95% CI)** | **Final Multivariable Model OR (95% CI)** |
| --- | --- | --- | --- |
| *Demographics* |  |  |  |
| Male Sex | 1.53 (0.67 - 3.49) | 1.37 (0.62 - 3.07) |  |
| Age, per year | 0.99 (0.97 - 1.00) | 0.98 (0.95 - 1.00) |  |
| Black Race | 0.81 (0.31 - 2.13) | 0.61 (0.22 - 1.70) |  |
| ICU location | 1.72 (0.85 - 3.50) | 0.92 (0.33 - 2.53) |  |
| Surgical Patient | 1.25 (0.63 – 2.49) | 1.87 (0.76 - 4.59) |  |
| *Laboratory Data* |  |  |  |
| Bicarbonate ≤ 24, per meq/L | **0.78 (0.73 - 0.84)^3^** | 0.92 (0.83 - 1.03) |  |
| Bicarbonate > 24, per meq/L | **0.75 (0.62 - 0.90)** | 0.93 (0.82 - 1.05) |  |
| BUN^5^, per 10 mg/dL | **1.32 (1.22 – 1.44)** | 1.07 (0.93 – 1.23) |  |
| Total calcium ≤9, per mg/dL | **0.53 (0.35 - 0.80)** | 0.65 (0.41 - 1.02) |  |
| Total calcium >9, per mg/dL | 0.52 (0.12 - 2.34) | 1.22 (0.34 - 4.41) |  |
| Creatinine, per mg/dL | **2.46 (1.93 - 3.12)** | **2.09 (1.54 - 2.83)** | **2.24 (1.81 - 2.78)** |
| Glucose ≤400, per 50 mg/dL | 1.16 (0.96 – 1.39) | 0.95 (0.76 – 1.20) |  |
| Glucose >400, per 50 mg/dL | **1.72 (1.16 – 2.55)** | **2.04 (1.24 – 3.35)** | **1.88 (1.22 – 2.90)** |
| Potassium ≤5, per meq/L | **1.85 (1.11 - 3.09)** | 1.12 (0.63 - 1.97) |  |
| Potassium >5, per meq/L | **1.77 (1.23- 2.57)** | 1.09 (0.41 - 2.90) |  |
| RDW^6^ ≤20, per 1% | 1.05 (0.92 - 1.21) | 1.00 (0.81 - 1.23) |  |
| RDW > 20, per 1% | 1.12 (0.97 - 1.29) | **1.18 (1.02 - 1.36)** | **1.20 (1.02 - 1.40)** |
| Sodium ≤140, per meq/L | **1.17 (1.02 - 1.34)** | 1.10 (0.98 - 1.23) |  |
| Sodium > 140, per meq/L | **1.12 (1.06 - 1.19)** | 1.04 (0.95 - 1.13) | **1.09 (1.02 - 1.16)** |
| Anion gap, per 1 unit | **1.21 (1.14 - 1.28)** | 0.96 (0.88 - 1.04) |  |
| Creatinine is increasing | **3.21 (2.32 - 4.43)** | **1.95 (1.44 - 2.64)** | **1.91 (1.39 - 2.63)** |
| Bun slope ≤0, per mg/dl/24h | 1.03 (0.97 - 1.09) | 1.00 (0.97 - 1.03) |  |
| Bun slope >0, per mg/dl/24h | **1.07 (1.05 - 1.09)** | **1.03 (1.02 - 1.05)** | **1.04 (1.03 - 1.05)** |
| *Medication Exposures^4^* |  |  |  |
| Pressors | **1.98 (1.06 - 3.68)** | 0.74 (0.30 - 1.82) |  |
| Narcotics | **0.31 (0.16 - 0.62)** | **0.27 (0.12 - 0.62)** | **0.33 (0.15 - 0.70)** |
| Paralytics | 2.36 (0.86 - 6.51) | 0.51 (0.11 - 2.41) |  |
| Total Parenteral Nutrition | **2.59 (1.30 - 5.16)** | 1.55 (0.61 - 3.93) |  |
| Loop diuretics | **2.09 (1.16 - 3.79)** | 1.68 (0.77 - 3.65) |  |
| Antibiotics | **2.71 (1.55 - 4.71)** | 1.85 (0.93 - 3.69) | **2.73 (1.44 - 5.18)** |

^1^ Covariates considered for inclusion in the conventional model

^2^ Odds ratios represent the time-updated likelihood of receiving RRT within 7 days, and data within 24-hrs of dialysis initiation was excluded.

^3^ Bold values are significant at a 2-sided p<0.05.

^4^ Medication exposures were defined as receiving a medication within the specified class prior to the time-point analyzed.

^5^ BUN= Blood Urea Nitrogen

^6^ RDW=Red Blood Cell Distribution Width
