# Supplementary material for: Approaches to Predicting Outcomes in Patients with Acute Kidney Injury
Source: PLoS One. 2017 Jan 25;12(1):e0169305. doi: 10.1371/journal.pone.0169305 (PMC5266278; doi:10.1371/journal.pone.0169305)
Supplement: S2 Table — (DOCX) [file pone.0169305.s004.docx]

**S2 Table – Conventional Model Predicting Death**

| **Table S2.** *Death ORs^1^: Before Dialysis Initiation* | | | |
| --- | --- | --- | --- |
| Covariate | Univariable Analysis  OR (95% CI^6^) | Full Multivariable Model OR (95% CI) | Final Multivariable Model OR (95% CI) |
| *Demographics* |  |  |  |
| Male Sex | 0.71 (0.40 - 1.26) | 0.62 (0.31 - 1.22) |  |
| Age, per year | 1.01 (0.98 - 1.03) | 1.02 (1.00 - 1.04) |  |
| Black Race | 0.83 (0.42 - 1.64) | 0.83 (0.38 - 1.83) |  |
| ICU^2^ location | 2.10 (1.15 - 3.82) ^*7^ | 1.14 (0.54 - 2.41) |  |
| Surgical Patient | 0.55 (0.30 - 1.00) ^*^ | 0.42 (0.20 - 0.90) ^*^ | 0.42 (0.21 - 0.85) ^*^ |
| *Laboratory Data* |  |  |  |
| Anion gap (per 1 unit) | 1.23 (1.17 - 1.31) ^*^ | 1.11 (1.01 - 1.23) ^*^ | 1.10 (1.04 - 1.15) ^*^ |
| Bicarbonate < 24 (per mEq/L) | 0.79 (0.74 - 0.85) ^*^ | 0.89 (0.78 - 1.02) | 0.88 (0.81 - 0.96) ^*^ |
| Bicarbonate ≥ 24 (per mEq/L) | 0.81 (0.69 - 0.96) ^*^ | 0.95 (0.83 - 1.10) |  |
| Bicarbonate Slope < 0 (per meq/L/24h) | 0.97 (0.95 - 0.99) ^*^ | 1.06 (1.01 - 1.11) ^*^ |  |
| Bicarbonate Slope ≥ 0 (per meq/L/24h) | 1.02 (0.99 - 1.05) | 1.01 (0.97 - 1.04) |  |
| BUN^3^ (per 10 mg/dl) | 1.02 (1.02 - 1.03) ^*^ | 1.23 (1.10 - 1.38) ^*^ | 1.01 (1.00 - 1.02) ^*^ |
| BUN slope < 0 (per mg/dl/24h) | 1.03 (0.97 - 1.09) | 1.03 (1.00 - 1.06) | 1.05 (1.01 - 1.09) ^*^ |
| BUN slope ≥ 0 & < 25 (per mg/dl/24h) | 1.10 (1.08 - 1.12) ^*^ | 1.02 (1.00 - 1.04) |  |
| BUN slope > 25 (per mg/dl/24h) | 1.04 (1.02 - 1.07) ^*^ | 0.97 (0.95 - 0.99) ^*^ |  |
| Total calcium < 9 (per mg/dl) | 0.39 (0.27 - 0.58) ^*^ | 0.60 (0.39 - 0.91) ^*^ | 0.50 (0.34 - 0.73) ^*^ |
| Total calcium ≥ 9 (per mg/dl) | 0.96 (0.41 - 2.26) | 2.19 (1.20 – 4.01) ^*^ | 2.02 (1.22 - 3.36) ^*^ |
| Chloride < 100 **(**meq/L**)** | 1.27 (1.04 - 1.54) ^*^ | 0.94 (0.79 - 1.13) |  |
| Chloride ≥ 100 (meq/L) | 1.12 (1.08 - 1.15) ^*^ | 1.02 (0.92 - 1.12) |  |
| Creatinine < 1 (per mg/dl) | 5.49 (0.39 - 76.5) | 0.19 (0.02 - 1.84) |  |
| Creatinine ≥ 1 & < 2 (per mg/dl) | 4.12 (2.16 - 7.86) ^*^ | 0.94 (0.38 - 2.34) |  |
| Creatinine > 2 (per mg/dl) | 1.15 (1.17 - 1.96) ^*^ | 0.74 (0.52 - 1.04) |  |
| Creatinine slope < -1 (per mg/dl/24h) | 1.11 (0.74 - 1.65) | 1.53 (0.85 - 2.77) |  |
| Creatinine slope ≥ -1 & < 0 (per mg/dl/24h) | 1.49 (0.71 - 3.12) | 1.16 (0.53 - 2.53) |  |
| Creatinine slope ≥ 0 & < 1 (per mg/dl/24h) | 6.36 (3.78 - 10.7) ^*^ | 1.59 (0.77 - 3.28) |  |
| Creatinine slope > 1 (per mg/dl/24h) | 1.63 (1.24 - 2.13) ^*^ | 1.17 (0.88 - 1.55) |  |
| Glucose < 200 (per 50 mg/dl) | 1.01 (1.00 - 1.01) | 1.08 (0.80 - 1.45) |  |
| Glucose ≥ 200 (per 50 mg/dl) | 1.00 (1.00 - 1.01) | 1.18 (0.94 - 1.48) |  |
| Glucose Slope < 0 (mg/dl) | 1.00 (1.00 - 1.00) | 1.00 (0.99 - 1.01) |  |
| Glucose Slope ≥ 0 (mg/dl) | 1.00 (1.00 - 1.00) | 1.01 (1.00 - 1.01) | 1.00 (1.00 - 1.00) ^*^ |
| Hemoglobin (g/dl) | 0.64 (0.53 - 0.77) ^*^ | 0.80 (0.68 - 0.94) ^*^ | 0.81 (0.68 - 0.97) ^*^ |
| Magnesium < 2.5 (meq/L) | 6.02 (2.51 - 14.5) ^*^ | 0.60 (0.25 - 1.44) |  |
| Magnesium ≥ 2.5 (meq/L) | 3.93 (0.93 - 16.5) | 0.30 (0.06 - 1.43) |  |
| MCV^4^ < 90 (fl) | 0.99 (0.93 - 1.05) | 0.98 (0.89 - 1.08) |  |
| MCV ≥ 90 (fl) | 0.93 (0.86 - 1.00) ^*^ | 0.93 (0.84 - 1.02) |  |
| Platelet Count < 200 (1000/ul) | 0.99 (0.99 - 1.00) ^*^ | 0.80 (0.63 - 1.02) |  |
| Platelet Count ≥ 200 (1000/ul) | 0.99 (0.99 - 1.00) ^*^ | 0.80 (0.65 - 1.00) | 0.99 (0.99 - 1.00) ^*^ |
| Potassium < 5 (per mEq/L) | 1.16 (0.75 - 1.78) | 1.25 (0.81 - 1.94) |  |
| Potassium > 5 (per mEq/L) | 2.46 (1.44 - 4.21) ^*^ | 1.96 (1.19 - 3.23) ^*^ | 2.24 (1.19 - 4.18) ^*^ |
| Potassium Slope < 0 (per mg/dl/24h) | 0.85 (0.79 - 0.91) ^*^ | 1.00 (0.91 - 1.11) |  |
| Potassium Slope ≥ 0 (per mg/dl/24h) | 1.20 (1.13 - 1.28) ^*^ | 1.04 (0.96 - 1.12) |  |
| RDW^5^ < 20 (per 1%) | 1.13 (1.01 - 1.26) ^*^ | 1.06 (0.89 - 1.25) |  |
| RDW ≥ 20 (per 1%) | 1.03 (0.92 - 1.16) | 0.94 (0.76 - 1.16) |  |
| Sodium < 140 (per mEq/L) | 1.39 (1.21 - 1.61) ^*^ | 1.12 (0.97 - 1.28) | 1.13 (1.02 - 1.24) ^*^ |
| Sodium ≥ 140 (per mEq/L) | 1.19 (1.14 - 1.25) ^*^ | 1.11 (0.99 - 1.24) | 1.10 (1.04 - 1.17) ^*^ |
| *Medication Exposures* |  |  |  |
| Pressors | 4.54 (2.78 - 7.42) ^*^ | 1.94 (0.99 - 3.78) | 1.94 (1.12 - 3.35) ^*^ |
| Narcotics | 0.40 (0.23 - 0.70) ^*^ | 0.60 (0.34 - 1.05) |  |
| Paralytics | 2.88 (1.26 - 6.60) ^*^ | 1.12 (0.50 - 2.51) |  |
| Total Parenteral Nutrition | 2.32 (1.26 - 4.24) ^*^ | 0.87 (0.42 - 1.77) |  |
| Loop diuretics | 1.46 (0.89 - 2.40) | 0.72 (0.40 - 1.30) |  |
| Antibiotics | 2.00 (0.84 - 4.79) | 0.97 (0.44 - 2.14) |  |

^1^ OR=odds ratio

^2^ ICU= intensive care unit

^3^ BUN= blood urea nitrogen

^4^ MCV= mean corpuscular volume

^5^ RDW= red cell distribution width

^6^ CI= confidence interval

^7^ *= p<0.05
