# Supplementary material for: Approaches to Predicting Outcomes in Patients with Acute Kidney Injury
Source: PLoS One. 2017 Jan 25;12(1):e0169305. doi: 10.1371/journal.pone.0169305 (PMC5266278; doi:10.1371/journal.pone.0169305)
Supplement: S3 Table — (DOCX) [file pone.0169305.s005.docx]

| **S3 Table.** *Death ORs^1^: After Dialysis Initiation* | | | |
| --- | --- | --- | --- |
| Covariate | Univariable Analysis  OR (95% CI^6^) | Full Multivariable Model OR (95% CI) | Final Multivariable Model OR (95% CI) |
| *Demographics* |  |  |  |
| Male Sex | 1.36 (0.45 - 4.05) | 2.04 (0.76 - 5.51) |  |
| Age, per year | 1.00 (0.97 - 1.04) | 0.99 (0.95 - 1.03) |  |
| Black Race | 0.50 (0.13 - 1.93) | 0.52 (0.18 - 1.51) |  |
| ICU^2^ location | 0.87 (0.36 - 2.05) | 1.19 (0.54 - 2.64) |  |
| Surgical Patient | 1.21 (0.46 - 3.15) | 2.71 (0.92 - 7.97) | 3.21 (1.19 - 8.68) ^*^ |
| *Laboratory Data* |  |  |  |
| Anion gap (per 1 unit) | 1.07 (1.00 - 1.14) ^*7^ | 1.32 (1.06 - 1.66) ^*^ | 1.26 (1.17 - 1.36) ^*^ |
| Bicarbonate < 24 (per mEq/L) | 0.78 (0.72 - 0.85) ^*^ | 0.91 (0.69 - 1.21) |  |
| Bicarbonate ≥ 24 (per mEq/L) | 0.52 (0.33 - 0.81) ^*^ | 0.97 (0.58 - 1.60) |  |
| Bicarbonate Slope < 0 (per meq/L/24h) | 0.94 (0.92 - 0.96) ^*^ | 1.01 (0.97 - 1.05) |  |
| Bicarbonate Slope ≥ 0 (per meq/L/24h) | 1.02 (1.00 - 1.05) | 1.02 (0.98 - 1.07) | 1.04 (1.00 - 1.07) ^*^ |
| BUN^3^ (per 10 mg/dl) | 1.00 (0.99 - 1.00) | 0.98 (0.81 - 1.19) |  |
| BUN slope < 0 (per mg/dl/24h) | 0.99 (0.99 - 1.00) | 1.00 (0.98 - 1.02) |  |
| BUN slope ≥ 0 & < 25 (per mg/dl/24h) | 0.98 (0.96 - 1.00) | 1.01 (0.98 - 1.05) |  |
| BUN slope > 25 (per mg/dl/24h) | 1.00 (0.97 - 1.03) | 1.01 (0.97 - 1.05) |  |
| Total calcium < 9 (per mg/dl) | 0.59 (0.37 - 0.92) ^*^ | 0.98 (0.54 - 1.77) |  |
| Total calcium ≥ 9 (per mg/dl) | 0.48 (0.12 - 1.95) | 0.31 (0.08 - 1.16) |  |
| Chloride < 100 (meq/L) | 1.12 (0.83 - 1.51) | 1.13 (0.84 - 1.52) | 1.28 (1.02 - 1.60) ^*^ |
| Chloride ≥ 100 (meq/L) | 1.02 (0.96 - 1.09) | 1.22 (1.01 - 1.48) ^*^ | 1.21 (1.10 - 1.33) ^*^ |
| Creatinine < 1 (per mg/dl) | 0.02 (0.00 - 2.39) | 1.27 (0.01 - 109) |  |
| Creatinine ≥ 1 & < 2 (per mg/dl) | 0.14 (0.05 - 0.36) ^*^ | 0.09 (0.02 - 0.38) ^*^ | 0.07 (0.02 - 0.22) ^*^ |
| Creatinine > 2 (per mg/dl) | 0.61 (0.41 - 0.92) ^*^ | 0.64 (0.36 - 1.13) |  |
| Creatinine slope < -1 (per mg/dl/24h) | 0.84 (0.71 - 0.99) ^*^ | 0.72 (0.57 - 0.92) ^*^ |  |
| Creatinine slope ≥ -1 & < 0 (per mg/dl/24h) | 0.64 (0.42 - 0.97) ^*^ | 1.32 (0.58 - 3.00) |  |
| Creatinine slope ≥ 0 & < 1 (per mg/dl/24h) | 0.51 (0.32 - 0.83) ^*^ | 1.05 (0.46 - 2.40) |  |
| Creatinine slope > 1 (per mg/dl/24h) | 0.76 (0.45 - 1.28) | 0.83 (0.54 - 1.26) |  |
| Glucose < 200 (per 50 mg/dl) | 1.00 (1.00 - 1.01) | 1.25 (0.87 - 1.79) |  |
| Glucose ≥ 200 (per 50 mg/dl) | 1.00 (1.00 - 1.01) | 0.80 (0.49 - 1.31) |  |
| Glucose Slope < 0 **(**per 50 mg/dl/24h) | 1.00 (1.00 - 1.00) ^*^ | 0.98 (0.97 - 0.99) ^*^ | 1.00 (1.00 - 1.00) ^*^ |
| Glucose Slope ≥ 0 (per 50 mg/dl/24h) | 1.00 (1.00 - 1.00) ^*^ | 1.00 (0.99 - 1.02) |  |
| Hemoglobin (g/dl) | 1.53 (1.18 - 1.98) ^*^ | 1.65 (1.18 - 2.29) ^*^ | 1.57 (1.19 - 2.07) ^*^ |
| Magnesium < 2.5 (meq/L) | 20.5 (6.67 - 62.7) ^*^ | 5.06 (1.39 - 18.4) ^*^ | 8.71 (2.45 - 31.0) ^*^ |
| Magnesium ≥ 2.5 (meq/L) | 2.46 (0.66 - 9.14) | 1.79 (0.20 - 15.6) |  |
| MCV^4^ < 90 (fl) | 0.96 (0.84 - 1.10) | 0.93 (0.71 - 1.24) |  |
| MCV ≥ 90 (fl) | 1.00 (0.91 - 1.11) | 1.08 (0.95 - 1.22) |  |
| Platelet Count < 200 (1000/ul) | 0.99 (0.98 - 0.99) ^*^ | 0.56 (0.33 - 0.94) | 0.99 (0.98 - 1.00) ^*^ |
| Platelet Count ≥ 200 (1000/ul) | 0.98 (0.96 - 1.00) | 0.78 (0.57 - 1.07) |  |
| Potassium < 5 (per mEq/L) | 3.97 (2.43 - 6.47) ^*^ | 1.47 (0.78 - 2.75) |  |
| Potassium > 5 (per mEq/L) | 8.72 (3.29 - 23.1) ^*^ | 2.34 (1.13 - 4.82) ^*^ | 2.99 (1.56 - 5.71) ^*^ |
| Potassium Slope < 0 (per mg/dl/24h) | 0.79 (0.70 - 0.88) ^*^ | 0.90 (0.77 - 1.06) |  |
| Potassium Slope ≥ 0 (per mg/dl/24h) | 1.29 (1.17 - 1.43) ^*^ | 1.06 (0.93 - 1.21) |  |
| RDW^5^ < 20 (per 1%) | 0.88 (0.71 - 1.09) | 0.84 (0.66 - 1.06) |  |
| RDW ≥ 20 (per 1%) | 1.06 (0.95 - 1.18) | 1.14 (0.95 - 1.37) |  |
| Sodium < 140 (per mEq/L) | 0.94 (0.86 - 1.04) | 0.74 (0.58 - 0.95) ^*^ | 0.70 (0.61 - 0.81) ^*^ |
| Sodium ≥ 140 (per mEq/L) | 0.97 (0.86 - 1.10) | 0.87 (0.71 - 1.07) |  |
| *Medication Exposures* |  |  |  |
| Pressors | 2.26 (0.82 - 6.22) | 1.62 (0.37 - 7.07) |  |
| Narcotics | 0.22 (0.10 - 0.48) ^*^ | 0.25 (0.11 - 0.57) ^*^ | 0.25 (0.11 - 0.56) ^*^ |
| Paralytics | 0.67 (0.25 - 1.76) | 1.79 (0.43 - 7.51) |  |
| Total Parenteral Nutrition | 1.20 (0.51 - 2.83) | 1.84 (0.62 - 5.52) |  |
| Loop diuretics | 1.24 (0.48 - 3.20) | 2.51 (0.91 - 6.98) |  |
| Antibiotics | 1.02 (0.21 - 5.02) | 0.18 (0.03 - 0.95) ^*^ |  |

^1^ OR=odds ratio

^2^ ICU= intensive care unit

^3^ BUN= blood urea nitrogen

^4^ MCV= mean corpuscular volume

^5^ RDW= red cell distribution width

^6^ CI= confidence interval

^7^ *= p<0.05
