# Supplementary material for: Approaches to Predicting Outcomes in Patients with Acute Kidney Injury
Source: PLoS One. 2017 Jan 25;12(1):e0169305. doi: 10.1371/journal.pone.0169305 (PMC5266278; doi:10.1371/journal.pone.0169305)
Supplement: S4 Table — (DOCX) [file pone.0169305.s006.docx]

**S4 Table – Traditional Model Predicting Length of Stay**

| **Table S4. Length of Stay: Before Dialysis Initiation** | | | |
| --- | --- | --- | --- |
| Covariate | Univariable Analysis Beta (95% CI^8^) | Full Multivariable Model Beta (95% CI) | Final Multivariable Model Beta (95% CI) |
| *Demographics* |  |  |  |
| Male Sex | 3.21 (0.26 - 6.16) *^9^ | 1.56 (-0.29 - 3.41) |  |
| Age, per year | -0.41 (-0.15 - 0.07) | -0.10 (-0.18 - -0.02) * | -0.12 (-0.20 - -0.03) * |
| Black Race | -2.49 (-5.90 - 0.91) | -0.03 (-2.69 - 2.62) |  |
| ICU^1^ location | 8.22 (5.39 - 11.0) * | 4.05 (1.83 - 6.28) * | 3.62 (1.53 - 5.71) * |
| Surgical Patient | 2.50 (-0.72 - 5.72) | 0.34 (-2.20 - 2.89) |  |
| *Laboratory Data* |  |  |  |
| Anion gap (per 1 unit) | 0.50 (-0.03 - 1.02) | 0.13 (-0.23 - 0.49) |  |
| Bicarbonate < 24 (per mEq/L) | -0.60 (-0.97- -0.23) * | -0.10 (-0.40 - 0.20) |  |
| Bicarbonate ≥ 24 (per mEq/L) | -0.35 (-1.08 - 0.38) | 0.01 (-0.67 - 0.69) |  |
| Bicarbonate Slope < 0 (per  meq/L/24h) | -0.34 (-0.49 - -0.19) * | -0.03 (-0.13 - 0.07) |  |
| Bicarbonate Slope ≥ 0 (per  mg/L/24h) | 0.25 (0.13 - 0.37) * | 0.01 (-0.08 - 0.10) |  |
| BUN^2^ (per 10 mg/dl) | 1.67 (0.98 - 2.37) * | 0.50 (0.02 - 0.98) * |  |
| BUN slope < 0 (per mg/dl/24h) | -0.11 (-0.22 - -0.01) * | 0.00 (-0.06 - 0.07) |  |
| BUN slope ≥ 0 (per mg/dl/24h) | 0.64 (0.35 - 0.94) * | 0.15 (0.02 - 0.28) * | 0.21 (0.06 - 0.36) * |
| Total calcium (per mg/dl) | -4.05 (-5.71 - -2.40) * | -1.17 (-2.39 - 0.04) | -1.23 (-2.44 - -0.01) * |
| Chloride < 100 (per meq/L) | 0.57 (0.20 - 0.94) * | -0.13 (-0.61 - 0.34) |  |
| Chloride ≥ 100 (per meq/L) | 0.73 (0.50 - 0.96) * | 0.00 (-0.28 - 0.29) |  |
| Creatinine (per mg/dl) | 2.45 (0.41 - 4.49) * | 1.32 (-0.23 - 2.86) | 2.41 (0.91 - 3.92) * |
| Creatinine slope < 0 (per mg/dl/24h) | -0.84 (-2.01 - 0.33) | 0.31 (-0.39 - 1.00) |  |
| Creatinine slope ≥ 0 (per mg/dl/24h) | 6.89 (2.28 - 11.5) * | 1.26 (-0.37 - 2.89) |  |
| Glucose < 200 (per 50 mg/dl) | 1.30 (0.22 - 2.37) * | -0.01 (-0.02 - 0.01) |  |
| Glucose ≥ 200 (per 50 mg/dl) | -0.63 (-1.38 - 0.13) | -0.01 (-0.02 - 0.01) |  |
| Glucose Slope < 0 (per 50  mg/dl/24h) | -0.11 (-0.16 - -0.06) * | 0.00 (0.00 - 0.00) |  |
| Glucose Slope ≥ 0 (per 50mg/dl/24h) | 0.04 (-0.01 - 0.08) | 0.00 (0.00 - 0.00) |  |
| Hemoglobin < 8g/dL (per g/dL) | -4.87 (-7.83 - -1.89) * | -1.38 (-3.29 - 0.53) |  |
| Hemoglobin ≥ 8g/dL (per g/dL) | -1.17 (-1.89 - -0.45) * | -0.25 (-0.85 - 0.36) |  |
| Magnesium < 2.5 (per meq/L) | 7.46 (2.36 - 12.6) * | 0.52 (-1.70 - 2.75) |  |
| Magnesium ≥ 2.5 (per meq/L) | 14.1 (-2.04 - 30.1) | 1.89 (-5.72 - 9.50) |  |
| MCH^3^ < 35 (per pg/cell) | 0.41 (-0.02 - 0.84) | 0.07 (-0.34 - 0.48) |  |
| MCH ≥ 35 (per pg/cell) | -0.57 (-1.39 - 0.26) | 0.42 (-0.69 - 1.53) |  |
| MCHC^4^ < 35 (per g/dL) | 1.21 (0.34 - 2.09) * | 0.48 (-0.40 - 1.36) | 0.77 (0.12 - 1.43) * |
| MCHC ≥ 35 (per g/dL) | 0.29 (-1.56 - 2.14) | -0.60 (-2.13 - 0.92) |  |
| MCV^5^ < 90 (per fL/cell) | 0.28 (-0.13 - 0.68) | 0.08 (-0.22 - 0.39) |  |
| MCV ≥ 90 (per fL/cell) | 0.00 (-0.21 - 0.21) | -0.07 (-0.34 - 0.20) |  |
| Platelet Count < 200 (per 50k/uL) | -1.29 (-2.31 - -0.27) * | -0.02 (-0.04 - 0.00) | -1.06 (-1.90 - -0.23) * |
| Platelet Count ≥ 200 (per 50k/uL) | -0.08 (-0.68 - 0.52) | 0.00 (0.00 - 0.01) |  |
| Potassium < 5 (per mEq/L) | -1.07 (-4.08 - 1.94) | 0.96 (-1.47 - 3.38) |  |
| Potassium > 5 (per mEq/L) | -0.82 (-2.55 - 0.90) | -1.66 (-3.27 - -0.04) * |  |
| Potassium Slope < 0 (per mg/dl/24h) | -1.68 (-2.15 - -1.21) * | -0.19 (-0.64 - 0.25) |  |
| Potassium Slope ≥ 0 (per mg/dl/24h) | 1.50 (1.02 - 1.99) * | 0.45 (0.16 - 0.74) * | 0.33 (0.00 - 0.66) * |
| RDW^6^ < 20 (per 1%) | 0.59 (-0.14 - 1.31) | 0.12 (-0.47 - 0.72) |  |
| RDW ≥ 20 (per 1%) | -0.32 (-0.68 - 0.04) | -0.06 (-0.47 - 0.35) |  |
| Sodium (per mEq/L) | 0.86 (0.61 - 1.11) * | 0.37 (0.05 - 0.70) * | 0.41 (0.24 - 0.58) * |
| WBC^7^ < 8k (per 1000/uL) | 0.03 (-0.53 - 0.60) | -0.43 (-0.92 - 0.07) |  |
| WBC ≥ 8k (per 1000/uL) | 0.11 (-0.09 - 0.31) | 0.06 (-0.04 - 0.15) |  |
| *Medication Exposures* |  |  |  |
| Pressors | 11.3 (7.96 - 14.6) * | 5.62 (2.54 - 8.70) * | 6.13 (3.27 - 8.99) * |
| Narcotics | -2.73 (-5.39 - -0.06) * | -2.34 (-4.20 - -0.47) * | -2.89 (-4.67 - -1.11) * |
| Paralytics | 11.7 (4.45 - 18.9) * | 2.46 (-2.24 - 7.16) |  |
| Total Parenteral Nutrition | 12.2 (8.00 - 16.4) * | 6.48 (3.14 - 9.82) * | 7.42 (4.19 - 10.7) * |
| Loop diuretics | 2.89 (0.54 - 5.24) * | -1.47 (-3.31 - 0.36) |  |
| Antibiotics | 4.33 (2.37 - 6.29) * | -1.33 (-3.02 - 0.36) |  |

^1^ ICU= intensive care unit

^2^ BUN= blood urea nitrogen

^3^ MCH= mean corpuscular hemoglobin

^4^ MCHC= mean corpuscular hemoglobin concentration

^5^ MCV= mean corpuscular volume

^6^ RDW= red cell distribution width

^7^ WBC= white blood cell

^8^ CI= confidence interval

^9^ *= p<0.05
