# Supplementary material for: Approaches to Predicting Outcomes in Patients with Acute Kidney Injury
Source: PLoS One. 2017 Jan 25;12(1):e0169305. doi: 10.1371/journal.pone.0169305 (PMC5266278; doi:10.1371/journal.pone.0169305)
Supplement: S5 Table — (DOCX) [file pone.0169305.s007.docx]

| **S5 Table. Length of Stay: After Dialysis Initiation** | | | |
| --- | --- | --- | --- |
| Covariate | Univariable Analysis Beta (95% CI^8^) | Full Multivariable Model Beta (95% CI) | Final Multivariable Model Beta (95% CI) |
| *Demographics* |  |  |  |
| Male Sex | 10.8 (-5.38 - 26.9) | 2.65 (-3.93 - 9.22) |  |
| Age, per year | -0.44 (-1.18 - 0.30) | -0.24 (-0.58 - 0.09) |  |
| Black Race | -1.29 (-21.6 - 19.0) | 6.45 (-7.69 - 20.60) |  |
| ICU^1^ location | 14.7 (0.47 - 29.0) *^9^ | 0.44 (-10.16 - 11.03) |  |
| Surgical Patient | 9.54 (-9.25 - 28.3) | -4.44 (-15.99 - 7.11) |  |
| *Laboratory Data* |  |  |  |
| Anion gap (per 1 unit) | -0.10 (-1.04 - 0.85) | 2.93 (-3.44 - 9.29) |  |
| Bicarbonate < 24 (per meq/L) | 1.25 (-0.84 - 3.34) | 4.26 (-2.19 - 10.72) |  |
| Bicarbonate ≥ 24 (per meq/L) | 5.51 (4.21 - 6.82) * | 5.10 (-1.61 - 11.81) | 3.18 (1.77 - 4.58) * |
| Bicarbonate Slope < 0 (per  meq/L/24h) | -0.40 (-0.86 - 0.05) | -0.17 (-0.48 - 0.14) |  |
| Bicarbonate Slope ≥ 0 (per  meq/L/24h) | 0.36 (0.07 - 0.64) * | -0.23 (-0.41 - -0.05) * |  |
| BUN^2^ (per 10 mg/dl) | 1.43 (0.06 - 2.80) * | 0.25 (-0.34 - 0.84) |  |
| BUN slope < 0 (per mg/dl/24h) | -0.02 (-0.23 - 0.19) | 0.04 (-0.03 - 0.11) |  |
| BUN slope ≥ 0 (per mg/dl/24h) | 0.24 (0.05 - 0.43) * | 0.14 (-0.05 - 0.32) |  |
| Total calcium (per mg/dl) | -8.16 (-15.3 - -0.99) * | -3.08 (-6.81 - 0.65) |  |
| Chloride < 100 (per meq/L) | 0.65 (-0.57 - 1.88) | 2.59 (-4.03 - 9.21) |  |
| Chloride ≥ 100 (per meq/L) | -0.20 (-1.45 - 1.06) | 3.52 (-2.84 - 9.89) |  |
| Creatinine (per mg/dl) | -0.83 (-4.19 - 2.52) | -0.45 (-2.25 - 1.35) |  |
| Creatinine slope < 0 (per mg/dl/24h) | -0.24 (-2.91 - 2.43) | -1.39 (-2.25 - -0.54) * |  |
| Creatinine slope ≥ 0 (per mg/dl/24h) | 0.85 (-3.05 - 4.74) | 0.46 (-1.52 - 2.43) |  |
| Glucose < 200 (per 50 mg/dl) | 1.10 (-4.30 - 6.49) | -0.02 (-0.07 - 0.04) |  |
| Glucose ≥ 200 (per 50 mg/dl) | -9.28 (-13.8 - -4.78) * | -0.06 (-0.10 - -0.02) * | -2.86 (-4.46 - -1.27) * |
| Glucose Slope < 0 (per | 0.05 (-0.18 - 0.28) | 0.00 (0.00 - 0.00) |  |
| Glucose Slope ≥ 0 **(**per 50 mg/dl/24h) | 0.09 (-0.12 - 0.30) | 0.00 (0.00 - 0.00) |  |
| Hemoglobin < 8 (per g/dL) | -12.0 (-20.4 - -3.63) * | -2.94 (-6.31 - 0.42) |  |
| Hemoglobin ≥ 8 (per g/dL) | -5.99 (-10.8 - -1.20) * | -0.40 (-2.36 - 1.55) |  |
| Magnesium < 2.5 (per mg/dL) | 20.2 (7.90 - 32.6) * | 1.89 (-5.04 - 8.83) |  |
| Magnesium ≥ 2.5 (per mg/dL) | 4.44 (-13.8 - 22.7) | 3.67 (-2.82 - 10.16) |  |
| MCH^3^ < 35 (per pg/cell) | -1.92 (-5.55 - 1.72) | 0.91 (-0.62 - 2.45) |  |
| MCH ≥ 35 (per pg/cell) | -6.06 (-9.53 - -2.60) * | -3.50 (-10.88 - 3.87) |  |
| MCHC^4^ < 35 (per pg/cell) | 3.47 (-4.70 - 11.6) | -0.19 (-1.86 - 1.48) |  |
| MCHC ≥ 35 (per pg/cell) | -0.62 (-17.2 - 16.0) | -9.51 (-20.03 - 1.02) |  |
| MCV^5^ < 90 (per fL/cell) | -1.66 (-5.36 - 2.05) | -0.87 (-2.00 - 0.27) |  |
| MCV ≥ 90 (per fL/cell) | -1.07 (-2.26 - 0.12) | 0.90 (0.19 - 1.61) * |  |
| Platelet Count < 200 (per 50k/uL) | -1.65 (-6.07 - 2.76) | -0.09 (-0.14 - -0.04) * | -3.71 (-6.24 - -1.17) * |
| Platelet Count ≥ 200 (per 50k/uL) | 1.79 (-2.77 - 6.36) | 0.02 (0.00 - 0.04) |  |
| Potassium < 5 (per mEq/L) | 10.84 (-2.85 - 24.5) | 2.53 (-4.22 - 9.27) |  |
| Potassium > 5 (per mEq/L) | 6.90 (-1.34 - 15.1) | 2.58 (-0.05 - 5.20) |  |
| Potassium Slope < 0 (per mg/dl/24h) | -2.68 (-3.90 - -1.46) * | -0.01 (-0.99 - 0.96) |  |
| Potassium Slope ≥ 0 (per mg/dl/24h) | 2.25 (0.48 - 4.03) * | 0.40 (-0.34 - 1.15) |  |
| RDW^6^ < 20 (per 1%) | -2.50 (-8.51 - 3.51) | 0.16 (-1.59 - 1.91) |  |
| RDW ≥ 20 (per 1%) | -2.17 (-4.05 - -0.29) * | -2.16 (-3.64 - -0.67) * | -1.94 (-3.62 - -0.26) * |
| Sodium (per mEq/L) | 0.65 (-0.56 - 1.86) | -2.91 (-9.27 - 3.45) |  |
| WBC^7^ < 8 (per thousand / uL) | 5.41 (2.05 - 8.77) * | 0.48 (-1.04 - 2.00) |  |
| WBC ≥ 8 (per thousand / uL) | 1.40 (0.87 - 1.93) * | 0.60 (0.30 - 0.91) * | 0.75 (0.31 - 1.19) * |
| *Medication Exposures* |  |  |  |
| Pressors | 21.2 (9.75 - 32.7) * | 12.02 (3.08 - 20.96) * |  |
| Narcotics | 3.70 (-5.31 - 12.7) | -4.90 (-9.65 - -0.16) * |  |
| Paralytics | 26.8 (10.2 - 43.4) * | 16.24 (4.85 - 27.63) * | 15.9 (3.09 - 28.7) * |
| Total Parenteral Nutrition | 23.7 (9.98 - 37.5) * | 11.58 (-0.98 - 24.15) | 15.8 (3.15 - 28.4) * |
| Loop diuretics | 2.01 (-17.1 - 21.1) | 4.10 (-3.05 - 11.25) |  |
| Antibiotics | 15.4 (2.69 - 28.2) * | -9.71 (-17.09 - -2.33) * |  |

^1^ ICU= intensive care unit

^2^ BUN= blood urea nitrogen

^3^ MCH= mean corpuscular hemoglobin

^4^ MCHC= mean corpuscular hemoglobin concentration

^5^ MCV= mean corpuscular volume

^6^ RDW= red cell distribution width

^7^ WBC= white blood cell

^8^ CI= confidence interval

^9^ *= p<0.05
